# Automated biphasic morphological assessment of hepatitis B-related liver fibrosis using second harmonic generation microscopy

**DOI:** 10.1038/srep12962

**Published:** 2015-08-11

**Authors:** Tong-Hong Wang, Tse-Ching Chen, Xiao Teng, Kung-Hao Liang, Chau-Ting Yeh

**Affiliations:** 1 Tissue Bank, Chang Gung Memorial Hospital, Tao-Yuan, Taiwan; 2 Department of Anatomic Pathology, Chang Gung Memorial Hospital, Chang Gung University School of Medicine, Tao-Yuan, Taiwan; 3 HistoIndex Pte Ltd, Singapore; 4 Liver Research Center, Department of Hepato-Gastroenterology, Chang Gung Memorial Hospital, Tao-Yuan, Taiwan

## Abstract

Liver fibrosis assessment by biopsy and conventional staining scores is based on histopathological criteria. Variations in sample preparation and the use of semi-quantitative histopathological methods commonly result in discrepancies between medical centers. Thus, minor changes in liver fibrosis might be overlooked in multi-center clinical trials, leading to statistically non-significant data. Here, we developed a computer-assisted, fully automated, staining-free method for hepatitis B-related liver fibrosis assessment. In total, 175 liver biopsies were divided into training (n = 105) and verification (n = 70) cohorts. Collagen was observed using second harmonic generation (SHG) microscopy without prior staining, and hepatocyte morphology was recorded using two-photon excitation fluorescence (TPEF) microscopy. The training cohort was utilized to establish a quantification algorithm. Eleven of 19 computer-recognizable SHG/TPEF microscopic morphological features were significantly correlated with the ISHAK fibrosis stages (P < 0.001). A biphasic scoring method was applied, combining support vector machine and multivariate generalized linear models to assess the early and late stages of fibrosis, respectively, based on these parameters. The verification cohort was used to verify the scoring method, and the area under the receiver operating characteristic curve was >0.82 for liver cirrhosis detection. Since no subjective gradings are needed, interobserver discrepancies could be avoided using this fully automated method.

Hepatitis B is a major infectious disease in Asia. Patients with chronic hepatitis B carry high liver cirrhosis and liver cancer risks^1^,^2^. Before the development of antiviral therapies, there was no efficient strategy for preventing chronic hepatitis B from progressing to end-stage liver disease^3^,^4^. According to reports from the World Health Organization, 2 billion people are estimated to have experienced a hepatitis B virus (HBV) infection during their lifetime, and more than 350 million are chronically infected. In addition, approximately 600,000 people die annually from acute or chronic HBV infection sequelae.

Liver fibrosis develops in patients with chronic hepatitis B because of repeated necroinflammation, followed by hepatocyte regeneration and the accumulation of extracellular matrix (ECM) proteins, such as collagen^5^,^6^. When hepatocytes are injured, the immune system is activated, and growth factors are released that activate hepatic stellate cells to produce collagen and other fibrillar components^7^. These substances are deposited into the ECM space. During liver injury, the collagen degradation process is impaired, which further leads to excessive ECM protein accumulation^8^. The end stage of liver fibrosis is liver cirrhosis, which can lead to liver cancer or liver failure.

Liver biopsy examinations are considered the “gold standard” for liver fibrosis diagnosis, by which clinical strategies for patient management are drafted. Biopsy samples provide direct information regarding the degrees of liver injury and fibrosis. Descriptive or semi-quantitative scoring systems are used for staging the disease extent based on liver histology findings. The traditional method classifies liver biopsy samples as absent, mild, moderate, or severe fibrosis. The first semi-quantitative scoring system, which was introduced in the 1980s, comprised a number range to indicate different categories of pathological features classified based on severity^9^. Several routinely used scoring systems were subsequently developed, including the Knodell histological activity index (HAI)^10^, Scheuer^11^, ISHAK^12^, and METAVIR^13^ systems. However, the pathological features that must be assessed using these systems do not have strict objective definitions. Thus, grading and staging scores are to some extent based on the subjective opinions of pathologists. Inter- and intra-observer variations can be as high as 35%, and obtaining highly reproducible results using these systems is difficult^14^,^15^,^16^.

Several studies have quantified liver fibrosis by employing image analysis systems^17^,^18^,^19^. These computer-aided systems are used to provide objective quantitative measurements that might exclude observer discrepancies. However, the staining-dependent processes used in these systems cause difficulties due to variations in the staining procedure between biopsy samples. Moreover, most of these systems define the liver fibrosis staging index only according to the total fibrosis area in the samples^20^,^21^,^22^. Other pathological features also play vital roles in liver fibrosis grading and staging^23^, necessitating computer-based systems that can provide measurements in addition to the total fibrosis area. Matalka *et al.*^23^ developed the first quantification system based on a pattern classification method; however, the features extracted from the images did not provide histopathological definitions. The system developed by Dioguardi^24^ permitted more than 10 quantitative measurements; however, these measurements were incompatible with those used by pathologists, and poor correlations were found between the obtained measurements and commonly used scoring systems. Goodman *et al.*^25^ summarized the common histopathologic features of liver biopsies that can be considered in staging systems (primarily morphological features). However, incorporating all of these particular morphological features into a pathologist-readable system for clinical use is challenging.

Recently, a staining-free approach that utilizes second harmonic generation (SHG) and two-photon excitation fluorescence (TPEF) microscopy has been developed, allowing for a more quantitative method to assess fibrosis^26^,^27^. By eliminating the use of staining, this method reduces the variations commonly observed in the staining process. Furthermore, the use of SHG provides a true quantitative measurement of the collagen level in the absence of sample digestion, leading to preservation of the morphological information in images^26^,^27^. Collagen quantitation using SHG/TPEF microscopy has been evaluated in animal models but not in a large collection of human tissues with various stages of fibrosis^26^,^27^. Such studies should be completed before this technique can be applied to patient samples.

Using both monophasic and biphasic models to interpret a biological system is not uncommon; for example, biphasic waveforms are commonly observed in electrocardiograms^28^,^29^. Many automated external defibrillators are designed to incorporate this biphasic nature for defibrillation with different electric waves at different times^30^. In this study, we used a similar biphasic approach to create differing models for the early and late stages of fibrosis development. We measured and quantified nineteen morphological features of collagen using SHG and TPEF microscopy and developed two algorithms for assessing fibrosis using these nineteen parameters for both early and later fibrosis stages. The results were validated with the histopathological ISHAK fibrosis assessment and conventional collagen staining methods, followed by transmitted light microscopy imaging. The validation results showed that the multivariate generalized linear model was better for assessing the fibrosis progression pattern at late liver fibrosis stages, whereas a non-linear (support vector machine, SVM) approach was better for the assessment of early fibrosis stages.

## Results

### Advanced SHG/TPEF microscopic quantification of fibrillar collagen was highly correlated with ISHAK fibrosis scores

To elucidate the SHG/TPEF microscopy imaging capacity for liver fibrosis, 175 liver biopsy samples were obtained from chronic HBV-infected patients. The clinical parameters of the recruited patients are listed in Table 1. One hundred and five randomly selected samples were assigned to the training cohort and used for developing and optimizing the computer-based algorithm for liver fibrosis quantification. The other 70 samples were used as a validation set to assess the performances of the models.

**Figure 1:**
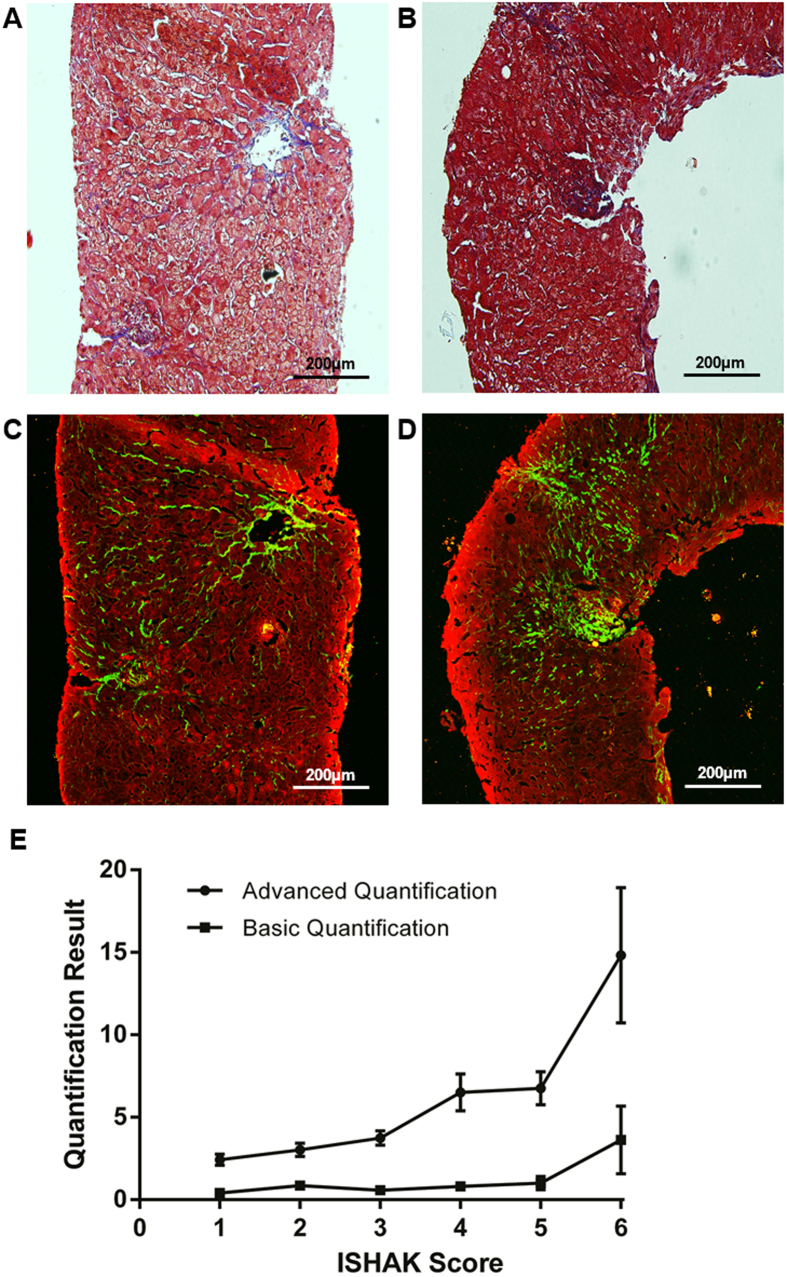
Comparison between SHG/TPEF imaging and linear microscopy after Masson’s trichrome staining. (**A**) and (**B**) represent fibrotic liver sections stained with Masson’s trichrome. Collagen fibers are stained in blue, and hepatocytes are stained in red. (**C**) and (**D**) are SHG/TPEF microscopy images. Fibrillar collagen (green) was detected by an SHG signal, and hepatocyte morphology (red) was observed with TPEF. (**E**) Correlations between the ISHAK scores and SHG/TPEF microscopy quantification results of samples from the training cohort. The total collagen levels are represented by solid squares, and the advanced collagen readings are represented by solid circles.

Figure 1A–D shows the images acquired using the conventional staining (Fig. 1A,B) and SHG/TPEF microscopy (Fig. 1C,D) methods. In SHG/TPEF imaging, fibrillar collagen was detected by SHG and is shown in green, whereas hepatocyte morphology was captured by TPEF microscopy and is shown in red. Visually, the SHG/TPEF technology resulted in the generation of a much more enhanced image with a better collagen distribution contrast. To minimize interference with normal-structured parenchyma and portal tract collagen, normal collagen readings were excluded. Figure 1E depicts the correlations identified by basic quantification (total collagen) or advanced quantification (Fibro-C-Index)^27^, excluding the normal-structured collagen signals^31^, with the ISHAK scores. The advanced quantification results were highly correlated with the ISHAK fibrosis scores.

### Morphological liver fibrosis features visualized by SHG/TPEF microscopy

**Figure 2:**
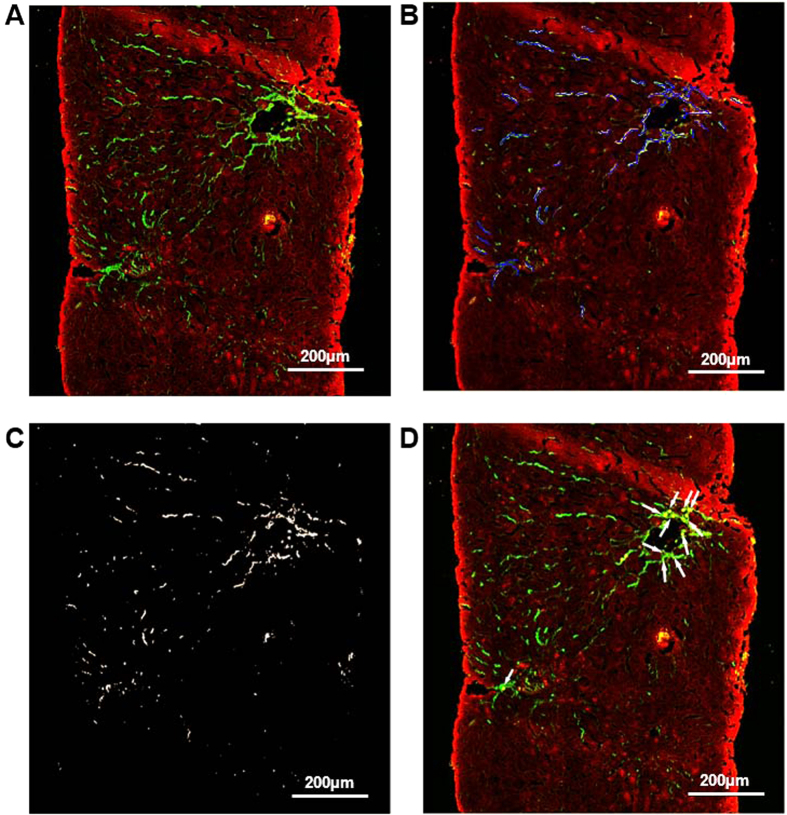
Morphological features used for qualitative liver fibrosis imaging. The total liver collagen ((**A**), green), different collagen strings ((**B**), colored region), collagen percentage of the total area (**C**), and crosslinked collagen ((**D**), white arrow) are shown.

**Figure 3:**
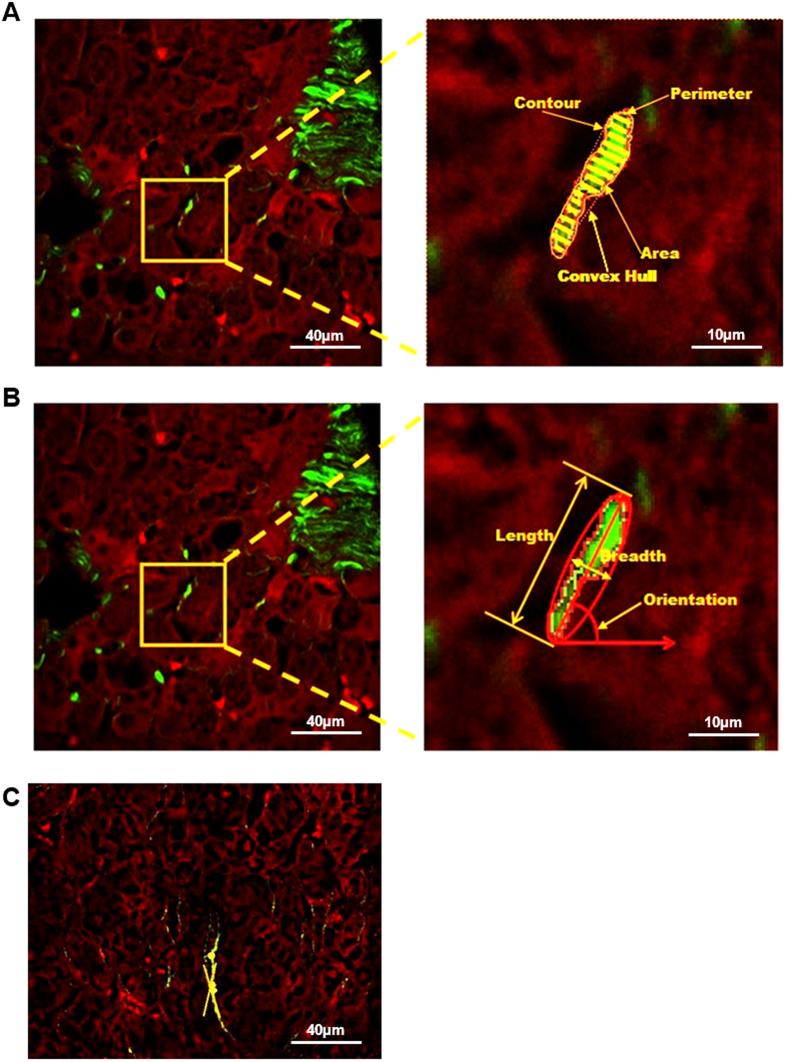
Primary morphological structural properties. Details of the morphological features, including the area, contour perimeter, convex hull, length, breadth, and collagen crosslinks, are shown in (**A–C**).

With the SHG/TPEF system, liver fibrosis severity could be quantitatively measured by examining the collagen fiber amount and morphology. Figure 2 shows the collagen profiles obtained from liver fibrosis qualitative imaging of the liver biopsies, including the total collagen distribution (A, green), various collagen strings (B, colored region), collagen percentage of the total area (CPA) (C), and collagen crosslinks (D, white arrow). The SHG/TPEF system employed 19 features (Table 2) to quantitatively measure liver fibrosis severity. These features were classified into three categories. The first feature category (F1-3) was collagen percentage, which included the total, aggregated and distributed collagen percentages. The second feature category (F4-13) was based on collagen string detection. Once a collagen string was detected, the contour that enclosed the collagen pixels was defined. Subsequently, the convex hull and the area and perimeter of each collagen string contour were also measured (Fig. 3A). To further describe the shapes and properties of the detected collagen strings, for each string, an ellipse was applied that enclosed all of the pixels used to calculate the global parameters (e.g., breadth and length) (Fig. 3B). Collagen connectivity (Fig. 3C) within the strings was calculated by skeletonizing them and then detecting the intersections between them.

The last set of features (F14-19) was generated according to the ratios of the different collagen string types.

### Correlation between the morphological features observed with SHG/TPEF microscopy and ISHAK fibrosis stages

To identify the critical morphological features that can be used to assess liver fibrosis, the correlations between the 19 morphological features and ISHAK fibrosis scores were analyzed using univariate linear regression. Assuming that the ISHAK fibrosis scores were a linear function that increased from 1 to 6, we calculated the P values of all 19 features. The results are shown in Table 2. Note that most of the first and second category features (11 of 13 features) were highly correlated (P < 0.001), whereas only some of the third category features were correlated (P < 0.05).

### Biphasic algorithm development for liver fibrosis assessment

In statistical analysis, collagen percentages (F1-3) were first employed to perform model fitting according to the ISHAK fibrosis scores (scores 1–6). Second, the collagen string properties (F4-13) were added. Finally, the collagen string ratios (F14-19) were included. A receiver operating characteristic (ROC) curve was used to assess the performance of the SHG/TPEF fibrosis score, using samples from the verification cohort (Table 3).

Overall, the sophisticated non-linear SVM model was superior to the generalized linear model. In both models, the additional morphological features significantly increased the area under the ROC curve (AUC). Additionally, some features were more important for identifying different stages, e.g., the F4-13 string properties in the SVM model were used to identify early-stage progression, and the F14-19 string ratios in the generalized linear model were used to identify late-stage progression.

Most combinations possessed high AUC values (most were higher than 0.7, but some were higher than 0.8), indicating that these features were crucial indices that were highly compatible with the clinically accepted ISHAK scores (i.e., these SHG-detected morphological features might facilitate efficient liver fibrosis assessments).

Among all of the models shown in Table 3, the largest AUC was measured while distinguishing between the non-cirrhotic (ISHAK stages 1–4) and cirrhotic (ISHAK 5–6) patients with the multivariate generalized linear model, which utilized all 19 included features (AUC = 0.829). Additionally, the generalized linear model was better at distinguishing early from advanced cirrhosis patients (12345 vs. 6; AUC = 0.826). However, the SVM model was optimal for classifying the early fibrotic stages (AUCs = 0.745, 0.774 and 0.797 for 1 vs. 2–6, 1–2 vs. 3–6 and 1–3 vs. 4–6, respectively). By utilizing the strengths of both model types, a biphasic strategy was proposed, the first step of which was to classify patients as either cirrhotic or non-cirrhotic using the generalized linear model with all 19 variables. If cirrhosis was detected, the same model was used for the score calculation. Otherwise, the SVM model was used to assess patients with early stage fibrosis.

## Discussion

Liver biopsy remains the standard for monitoring fibrosis progression, in which a small piece of liver tissue is removed using a biopsy needle, stained, examined under a microscope, and graded based on a descriptive or semi-quantitative scoring method^32^. In addition to the potential complications related to the biopsy procedure, inherent drawbacks exist, including potentials for sampling errors, staining variations, and inter- and intra-observer variabilities, in the interpretation of histology results^32^,^33^.

With the development of both mode-locked lasers and highly sensitive optical sensors, non-linear optical microscopy, such as multi-photon excitation fluorescence and multi-harmonic generation, has become an affordable option for tissue imaging^34^,^35^. Because TPEF microscopy has a different excitation mechanism than SHG, TPEF signals can be easily separated from SHG signals using the appropriate detectors. Combined TPEF/SHG images reveal additional fibrotic liver data, such as precise collagen distributions and undistorted liver cell morphologies. In recent studies, TPEF microscopy has been widely used for imaging organ structure components and their dynamic interactions in biological tissues. Additionally, SHG microscopy has been increasingly used for measuring highly ordered structures without central symmetry, such as type I collagen, which is the dominant collagen type in fibrotic livers^36^,^37^,^38^.

Compared with conventional collagen imaging, which uses transmitted light microscopy of histological tissue sections that are stained with either Masson’s trichrome or picrosirius red, SHG microscopy has several advantages, as follows: (1) this method provides superior information regarding fibrillar collagen; (2) sample staining is unnecessary; thus, the staining variations that result from different stain batches, protocols, time-dependent fading, and photobleaching^26^,^39^ are eliminated; (3) fluorophores are absent from tissues; thus, signals are unaffected by dye concentrations and photobleaching; (4) infrared excitation sources can be used, resulting in less scattering in the tissue; and finally, (5) SHG microscopy enables deeper tissue penetration for imaging purposes; hence, the 3D visualization of fiber architecture can be achieved^40^. Additionally, TPEF microscopy can provide complementary structural and cellular information^41^.

Fibrillar collagen formation occurs late in fibrogenesis. A traditional fibrosis microscopy evaluation is commonly based on restricted assessments related to later fibrotic changes, whereas features of the early stages of fibrogenesis are usually neglected. However, the use of new morphological criteria in conjunction with standard histopathological fibrosis scoring could improve the assessments of relatively small amounts of fibrillar collagen within fibrous tissues. This study evaluated 19 morphological features of SHG/TPEF images that are automatically included by the Genesis program. Morphological features such as collagen string formation and connectivity may allow for the identification of several stages in the progression of fibrillar collagen formation; therefore, these morphological features may enable the evaluation of early stages of fibrogenesis. Gailhouste *et al.*^42^ have highlighted the benefits of the linear scale provided by the SHG index by comparing it with the histological METAVIR scoring system. However, they have shown that the continuous scale provides greater accuracy than the semi-quantitative scoring system because it underestimates variables that demonstrate architectural differences in collagen, resulting in the restricted SHG indexing of fibrosis stages.

In this study, a biphasic model was developed to separately assess the early and late stages of fibrosis. We compared a multivariate generalized linear model with the SVM model. The results showed that the SVM approach was superior for detecting and differentiating between fibrosis stages (ISHAK 1–4), suggesting that collagen accumulation during fibrosis progression might not be a linear process. The finding that the biphasic approach displayed better accuracy than the monophasic approach further suggests that the pace of collagen accumulation differs during the early and late stages of fibrosis development. After incorporating the SVM into the biphasic approach, we successfully achieved an ROC of >0.82 for monitoring late liver fibrosis stages (cirrhosis) and an ROC of >0.75 for monitoring early liver fibrosis stages. We also observed that the addition of many parameters with relatively high P values (P > 0.001) resulted in better performance for assessing the fibrosis degree, especially for the non-linear models. For example, F14-19, which includes the different collagen string feature ratios, was not directly correlated with the degree of fibrosis; however, the addition of these parameters to the multivariate generalized linear model resulted in significant improvements in the ROC values in most cases.

AUC analysis revealed that the inclusion of some features, such as the string properties (F4-13), into the SVM models helped to improve the distinction of early stage fibrosis stage, whereas including the string ratios (F14-19) in the generalized linear models helped to improve late fibrosis stage distinction. The first part of these results is in agreement with the results of a previous study of early stage collagen remodeling^26^, which revealed alterations in collagen shapes but no substantial change in the total amount of collagen. Moreover, the second part of our findings suggests that although the total amount of collagen is altered during late liver fibrosis stages, the ratio of the different collagen string features is a better method for assessing fibrosis stages.

In this study, the main reason for liver biopsy of chronic hepatitis B patients was pre-treatment evaluation. Therefore, these patients were mostly in the immune clearance stage and were not evenly distributed over all clinical stages. As a result, many patients were positive for HBeAg but were already progressing to liver cirrhosis, suggesting a failure of HBeAg seroconversion despite repeated hepatitis flares. However, even during the immune clearance phase, patients who were positive for HBeAg still had a borderline lower fibrosis score (mean ± SD, 3.42 ± 1.41 vs. 3.06 ± 1.36; HBeAg negative vs. positive, P = 0.156).

In conclusion, this study developed a fully automated and quantitative assessment system employing SHG/TREF microscopy to specifically assess HBV-related fibrosis. We systematically optimized the parameters for quantitative SHG/TPEF liver tissue imaging and developed fully automated image analysis algorithms. Compared with the traditional staining systems, the present system enables enhanced collagen fiber detection and quantification for fibrosis research and clinical diagnosis. SHG/TPEF microscopy is a standard analytical tool, and its use in combination with a computer-based detection and calculation system represents a powerful technique that can be applied in multi-center comparisons of liver fibrosis.

## Methods

### Tissue Preparation

The study cohort consisted of 175 liver biopsy samples from chronic HBV-infected patients who underwent liver biopsies at LinKo Chang Gung Memorial Hospital between 2000 and 2012. Clinical and pathological characteristics were obtained from the patients’ medical records. As described below, liver fibrosis was staged by pathologists according to the ISHAK scoring system and was then automatically evaluated with computational algorithms according to 19 morphological variables. Of the 175 samples, 105 (training cohort) were used for developing a computer-based algorithm with a close correlation to the ISHAK fibrosis score. The other 70 samples were used to validate the scoring method derived from the training system.

This study was approved by the Ethics Committee of Chang Gung Memorial Hospital. Written informed consent was obtained from each patient, and the study was carried out in accordance with the approved guidelines.

### Masson’s Trichrome Staining

Masson’s trichrome staining was performed at the Chang Gung Memorial Hospital Department of Anatomic Pathology as follows. First, 5-micron FFPE sections were deparaffinized and hydrated in distilled water. Then, Bouin’s fixative was used as a mordant for 1 h at 56 °C. The FFPE sections were cooled and washed in running water until the yellow coloring disappeared. The samples were stained in Weigert’s hematoxylin stain for 10 min, thoroughly washed in tap water for 10 min, stained again in an acid fuchsin solution for 15 min, and then rinsed in distilled water for 3 min. After rinsing, the slides were treated with phosphomolybdic acid solution for 10 min and then rinsed in distilled water for 10 min. Finally, the slides were stained with a light-green solution for 2 min and rinsed in distilled water. After thorough dehydration using alcohol, the slides were mounted, and coverslips were placed onto them.

### Image Acquisition System

Images were acquired using a Genesis^TM^ (HistoIndex Pte. Ltd, Singapore) system, in which SHG microscopy was used to visualize collagen, and the other cell structures were visualized with TPEF microscopy. Details of the optical settings used can be obtained from Sun *et al.*^26^ and Tai *et al.*^27^.

The samples were laser-excited at 780 nm, SHG signals were recorded at 390 nm, and TPEF signals were recorded at 550 nm. Images were acquired at 20× magnification with 512 × 512 pixel resolution, and each image had a dimension of 200 × 200 μm. Multiple adjacent images were captured to encompass large areas. Nine images (3 × 3 images, 600 × 600 μm dimension) were acquired per site. Overall, five 3 × 3 images from five sites were obtained for each sample.

### SHG Quantification

Total collagen percentages and previously described collagen features, including specific collagen strings and collagen connectivity-related measurements, were used to assess correlations with the ISHAK fibrosis scores. Nineteen morphological features were used in this study. These variables were divided into three groups, as follows:

#### Group 1

CPA (collagen percentage of the total area), Agg (the aggregated collagen percentage of the total area), and Dis (the distributed collagen percentage of the total area). The total collagen level was detected by SHG and segmented from the raw images. The aggregated and distributed collagen were differentiated with a low-pass filter^26^.

#### Group 2

NoStr (collagen string number), NoShortStr (short collagen string number), NoLongStr (long collagen string number), NoThinStr (thin collagen string number), NoThickStr (thick collagen string number), StrArea (total string area), StrLength (total string length), StrWidth (total string width), StrPerimeter (total string perimeter), and NoXlink (total crosslink number). The collagen strings were detected using a connected-component algorithm of binary images from a previous image segmentation. Each connected component was fitted with an ellipse, and then the axes of the ellipses were used to divide the strings into long and short as well as thin and thick sub-groups. The total lengths, widths, areas and perimeters of the collagen strings were also measured. We used an image skeleton algorithm to detect branch points for determining crosslink numbers.

#### Group 3

NoShortStr/NoStr (ratio between the short string and total string numbers), NoLongStr/NoStr (ratio between the long string and total string numbers), NoShortStr/NoLongStr (ratio between the short string and long string numbers), NoThinStr/NoStr (ratio between the thin string and total string numbers), NoThickStr/NoStr (ratio between the thick string and total string numbers), and NoThinStr/NoThickStr (ratio between the thin string and thick string numbers). We found that these relative ratios improved the system only when absolute (normalized) values were used for Group 2.

### Statistical Analysis and Model Construction

Univariate analyses were performed to assess correlations between individual image features and the ISHAK scores using linear regression. Two modeling method types were used for the patient classifications, which were based on multiple image features. In the first method, the dependent variable, y, logit, which was a fibrosis stage indicator, was a linear combination of *n* feature variables *X*_*1*_ *… X*_*n*_, as shown in the following equation:


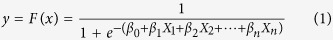


The coefficients *β*_0_, *β*_1_, …*β*_*n*_ were estimated with the maximum likelihood method.

The second method used was the SVM model. A hyperplane was constructed for optimally classifying the subjects into fibrosis stages based on the feature values in the *m-*dimensional space (*m* ≥ *n*). The radial basis function (RBF) kernel was used as the distance measure between two subject vectors *x* and *x’*, i.e.,


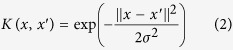


where σ controls the width of the kernel.

## Additional Information

**How to cite this article**: Wang, T.-H. *et al.* Automated biphasic morphological assessment of hepatitis B-related liver fibrosis using second harmonic generation microscopy. *Sci. Rep.* **5**, 12962; doi: 10.1038/srep12962 (2015).

## Figures and Tables

**Table 1:** Basic clinical data for the patients whose liver tissues were submitted for fibrosis evaluation.

	Training cohort	Verification cohort
	n = 105	n = 70
Age (years)	45.7 ± 11.8	44.0 ± 11.2
Male, n (%)	85 (83.3)	59 (84.3)
Biopsy		
Necroinflammation score		
Periportal inflammation	1.2 ± 0.7	0.9 ± 0.9
Confluence necrosis	0.1 ± 0.5	0.2 ± 0.9
Focal inflammation	2.0 ± 0.7	2.0 ± 0.8
Portal inflammation	2.4 ± 0.9	2.2 ± 1.0
ISHAK fibrosis score, n (%)		
1	12 (11.4)	10 (14.3)
2	19 (18.1)	12 (17.1)
3	27 (25.7)	24 (34.3)
4	15 (14.3)	10 (14.3)
5	22 (21.0)	11 (15.7)
6	10 (9.5)	3 (4.3)
HBeAg-positive, n (%)	37(52.1)	31 (63.3)
Biochemistry		
AST (IU/mL)	117.0 ± 92.0	92.6 ± 120.7
ALT (IU/mL)	220.9 ± 201.1	162.9 ± 202.5
AST/ALT	0.6 ± 0.4	0.6 ± 0.3
AFP (ng/mL)	10.5 ± 26.7	13.2 ± 65.7
Bilirubin (mg/dL)	1.1 ± 0.7	1.0 ± 0.4
GGT (IU/L)	80.4 ± 94.7	49.9 ± 43.6
Albumin (g/dL)	4.6 ± 0.3	4.5 ± 0.4
Platelets (×1000/mm^3^)	185.5 ± 58.0	189.8 ± 55.0
Prothrombin time prolongation (sec)	0.7 ± 0.6	1 ± 0.9
White blood cells (×1000/mm^3^)	5.6 ± 1.8	5.4 ± 1.5
Hemoglobin (g/dL)	15.0 ± 1.5	14.9 ± 1.5
Alpha-1 globulin (g/dL)	0.2 ± 0.1	0.2 ± 0.1
Alpha-2 globulin (g/dL)	0.8 ± 0.2	0.8 ± 0.1
Beta globulin (g/dL)	0.8 ± 0.2	0.8 ± 0.1
Gamma globulin (g/dL)	1.6 ± 0.4	1.5 ± 0.5
Albumin/globulin	1.5 ± 0.3	1.5 ± 0.4

**Table 2:** Univariate linear regression analysis of the 19 SHG morphological features (F1 to F19) and ISHAK fibrosis scores.

No.	Feature abbr.	Feature full name	All P value	Training cohort P value	Verification cohort P value
F1	Cpa	Collagen percentage of area	<0.0001	<0.0001	0.0022
F2	Agg	Aggregated collagen percentage of area	<0.0001	<0.0001	0.0020
F3	Dis	Distributed collagen percentage of area	0.2401	0.3254	0.5274
F4	NoStr	Number of collagen strings	<0.0001	<0.0001	<0.0001
F5	NoShortStr	Number of short collagen strings	<0.0001	<0.0001	<0.0001
F6	NoLongStr	Number of long collagen strings	<0.0001	<0.0001	<0.0001
F7	NoThinStr	Number of thin collagen strings	0.0001	0.0004	0.0372
F8	NoThickStr	Number of thick collagen strings	<0.0001	<0.0001	<0.0001
F9	StrArea	Total string area	<0.0001	<0.0001	0.0051
F10	StrLength	Total string length	<0.0001	<0.0001	<0.0001
F11	StrWidth	Total string width	<0.0001	<0.0001	<0.0001
F12	StrPerimeter	Total string perimeter	<0.0001	<0.0001	<0.0001
F13	NoXlink	Total number of crosslinks	<0.0001	0.0033	0.0004
F14	NoShortStr/NoStr	Ratio between the number of short strings and the total number of strings	0.0011	<0.0001	0.2511
F15	NoLongStr/NoStr	Ratio between the number of long strings and the total number of strings	0.0011	<0.0001	0.2511
F16	NoShortStr/NoLongStr	Ratio between the number of short strings and the number of long strings	0.0002	<0.0001	0.1689
F17	NoThinStr/NoStr	Ratio between the number of thin strings and the total number of strings	0.1276	0.8378	0.1008
F18	NoThickStr/NoStr	Ratio between the number of thick strings and the total number of strings	0.1276	0.8378	0.1008
F19	NoThinStr/NoThickStr	Ratio between the number of thin strings and the number of thick strings	0.0312	0.6115	0.0343

**Table 3:** Performances of the SHG/TPEF fibrosis scoring models (AUCs) (generalized linear model vs. SVM) using different combinations of morphological features.

ISHAK fibrosis stages	Features used for modeling					
	F1-3		F1-13		F1-19	
	AUC	P	AUC	P	AUC	P
Generalized Linear Model						
1 vs. 23456	0.595	0.34	0.597	0.33	0.640	0.16
12 vs. 3456	0.552	0.49	0.608	0.15	0.628	0.09
123 vs. 456	0.697	0.01	0.687	0.01	0.788	<0.01
1234 vs. 56	0.745	<0.01	0.788	<0.01	0.829	<0.01
12345 vs. 6	0.736	0.17	0.408	0.59	0.826	0.06
SVM (RBF kernel) model						
1 vs. 23456	0.587	0.38	0.735	0.02	0.745	0.01
12 vs. 3456	0.548	0.52	0.641	0.06	0.774	<0.01
123 vs. 456	0.697	0.01	0.813	<0.01	0.797	<0.01
1234 vs. 56	0.745	<0.01	0.795	<0.01	0.814	<0.01
12345 vs. 6	0.736	0.17	0.726	0.19	0.806	0.07
